# *miR-31* is consistently inactivated in EBV-associated nasopharyngeal carcinoma and contributes to its tumorigenesis

**DOI:** 10.1186/1476-4598-13-184

**Published:** 2014-08-07

**Authors:** Chartia Ching-Mei Cheung, Grace Tin-Yun Chung, Samantha Wei-Man Lun, Ka-Fai To, Kwong-Wai Choy, Kin-Mang Lau, Sharie Pui-Kei Siu, Xin-Yuan Guan, Roger Kai-Cheong Ngan, Timothy Tak-Chun Yip, Pierre Busson, Sai-Wah Tsao, Kwok-Wai Lo

**Affiliations:** 1 Department of Anatomical and Cellular Pathology, State Key Laboratory in Oncology in South China, Prince of Wales Hospital, The Chinese University of Hong Kong, Hong Kong, People’s Republic of China; 2 Li Ka Shing Institute of Health Science, The Chinese University of Hong Kong, Hong Kong, People’s Republic of China; 3 Department of Obstetrics and Gynecology, Prince of Wales Hospital, The Chinese University of Hong Kong, Hong Kong, People’s Republic of China; 4 Department of Clinical Oncology, University of Hong Kong, Hong Kong, People’s Republic of China; 5 Department of Clinical Oncology, Queen Elizabeth Hospital, Hong Kong, People’s Republic of China; 6 CNRS-UMR 8126 and Institut de cancérologie Gustave Roussy, Université Paris-Sud-11, 39 rue Camille Desmoulins, F-94805 Villejuif, France; 7 Department of Anatomy, University of Hong Kong, Hong Kong, People’s Republic of China

**Keywords:** Nasopharyngeal carcinoma, MicroRNA, miR-31, FIH1, MCM2

## Abstract

**Background:**

As a distinctive type of head and neck cancers, nasopharyngeal carcinoma (NPC) is genesis from the clonal Epstein-Barr virus (EBV)-infected nasopharyngeal epithelial cells accumulated with multiple genetic lesions. Among the recurrent genetic alterations defined, loss of 9p21.3 is the most frequent early event in the tumorigenesis of EBV-associated NPC. In addition to the reported *CDKN2A/p16*, herein, we elucidated the role of a miRNA, *miR-31* within this 9p21.3 region as NPC-associated tumor suppressor.

**Methods:**

The expression and promoter methylation of miR-31 were assessed in a panel of NPC tumor lines and primary tumors. Its *in vitro* and *in vivo* tumor suppression function was investigated through the ectopic expression of *miR-31* in NPC cells. We also determined the *miR-31* targeted genes and its involvement in the growth in NPC.

**Results:**

Downregulation of *miR-31* expression was detected in almost all NPC cell line, patient-derived xenografts (PDXs) and primary tumors. Both homozygous deletion and promoter hypermethylation were shown to be major mechanisms for *miR-31* silencing in this cancer. Strikingly, loss of *miR-31* was also obviously observed in the dysplastic lesions of nasopharynx. Restoration of *miR-31* in C666-1 cells inhibited the cell proliferation, colony-forming and migratory capacities. Dramatic reduction of *in v*itro anchorage-independent growth and *in vivo* tumorigenic potential were demonstrated in the stable clones expressing *miR-31*. Furthermore, we proved that *miR-31* suppressed the NPC cell growth via targeting FIH1 and MCM2.

**Conclusions:**

The findings provide strong evidence to support *miR-31* as a new NPC-associated tumor suppressor on 9p21.3 region. The inactivation of *miR-31* may contribute to the early development of NPC.

## Background

MicroRNAs, about 21–25 nucleotides in length, are endogenous non-coding RNAs that regulate gene expression negatively at post-transcriptional level [1,2]. Increasing evidence indicates that microRNAs can contribute to the tumorigenesis process by modulating various cellular mechanisms, such as proliferation, apoptosis, and cell migration and invasion. To date, a number of host- and virus-encoded microRNAs were demonstrated to be aberrantly expressed and play important roles in the development of human cancers [3,4].

Nasopharyngeal carcinoma (NPC) is an Epstein-Barr virus (EBV)-associated epithelial malignancy that is prevalent in Southern China and Southeast Asia. In addition to EBV infection, a number of recurrent genetic changes contribute to NPC multi-step tumorigenesis. Through comprehensive investigation of a panel of precancerous lesions and normal nasopharyngeal epithelia, we have previously demonstrated the occurrence of allelic loss on chromosome 3p and 9p prior to EBV latent infection during the initiation of NPC [5,6]. Inactivation of the key tumor suppressor genes on these regions such as *RASSF1A* (3p21.3) and *p16/CDKN2A* (9p21.3) were proven to be critical events in NPC tumorigenesis. Recently, we investigated the miRNA profiles of a panel of EBV-associated NPC tumor lines and identified several differentially expressed miRNAs that may contribute to NPC development. Among the aberrantly expressed miRNAs identified, the *miR-31*, which is located on the common homozygous deletion region on chromosome 9p21.3 and adjacent to the *p16/CDKN2A* locus, is consistently down-regulated in NPC [7]. Since down-regulation of *miR-31* contributes to the progression of prostate, ovarian, and breast cancers, we hypothesize that *miR-31* is one of the critical NPC-associated tumor suppressor on chromosome 9p and may involve in the early development of this cancer [8-10]. Herein, we revealed the mechanisms involved in the inactivation of *miR-31*, identified the direct targets and demonstrated its tumor suppressor function in NPC cells. Our study provides strong evidence that inactivation of *miR-31* in the 9p21.3 tumor suppressor loci is an important event in NPC tumorigenesis.

## Results

### Consistent down-regulation of miR-31 in NPC

**Figure 1:**
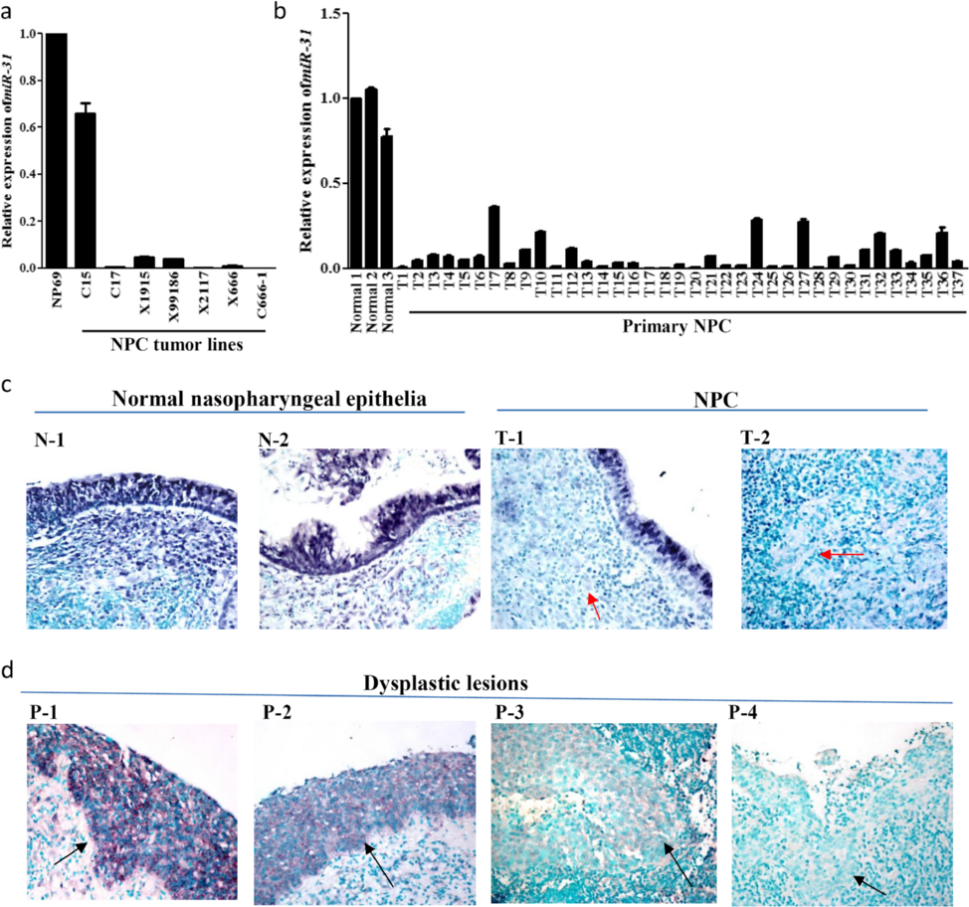
**Consistent down-regulation of *miR-31 *in NPC.** By quantitative RT-PCR, loss or high reduction of *miR-31* expression was detected in **(a)** a NPC cell line, 5/6 xenografts and **(b)** all 37 primary tumors. The immortalized normal nasopharyngeal epithelial cell line NP69 and microdissected normal epithelia (Normal 1–3) were included as controls. **(c)** Representative images of in-situ hybridization showing the high *miR-31* expression in normal nasopharyngeal epithelia and loss of *miR-31* expression in the NPC tumor cells (red arrow) (X400). **(d)** By in situ hybridization, loss of *miR-31* expression was found in 2/4 dysplastic lesions (black arrow) (X400).

In our earlier studies, homozygous deletion of 9p21.3 including the *CDKN2A/CDKN2B* loci was commonly found in EBV-associated NPC [11]. In addition to the well-known tumor suppressor function of *p16/CDKN2A*, it is suspected that inactivation of other candidate genes in this region may also contribute to the NPC tumorigenesis. *MiR-31*, which is at 0.5 Mb telomeric to the *CDKN2A/p16* loci, was shown to function as tumor suppressor microRNA in various human cancers [7,12,13]. Using microRNA microarray, we examined the microRNA expression profiles in the immortalized nasopharyngeal epithelial cell NP69 and a panel of NPC cell line and patient derived xenografts (PDXs). Hierarchical clustering with average linkage algorithm was performed and a heat map of the expression profiles was generated (Additional file 1: Figure S1). Among the 115 differentially expressed miRNAs identified, we noted that the *miR-31* expression was highly reduced in 5/6 NPC xenografts. This preliminary finding suggested the inactivation of *miR-31* is common in this EBV-associated cancer. To confirm the frequent down-regulation of *miR-31* in NPC, we have assessed its expression in a panel of tumor lines and microdissected primary tumors by stem-looped qRT-PCR. As shown in Figure 1A, *miR-31* expression was highly reduced in 5 of 6 (83.3%) EBV-positive xenografts and in all 37 (100%) primary tumors (Figure 1a and 1b). Down-regulation of *miR-31* was also detected in the EBV-positive NPC cell line C666-1 which is originally derived from xeno-666. Abundant *miR-31* transcription was only detected in the C15 xenograft which expresses EBV-encoded LMP1 protein (Figure 1a). In Figure 1c, *in-situ* hybridization analysis demonstrated the high *miR-31* expression in normal nasopharyngeal epithelia and down-regulation of *miR-31* in the tumor cells of representative cases. Importantly, down-regulation of *miR-31* was also obviously detected in 2/4 dysplastic lesions which we collected in our previous studies (Figure 1d) [14,15]. Our finding not only revealed the consistent inactivation of *miR-31* in EBV-associated NPC, it also provided first evidence for the involvement of *miR-31* down-regulation in the early development of NPC.

### Homozygous deletion and promoter hypermethylation of *miR-31*

**Figure 2:**
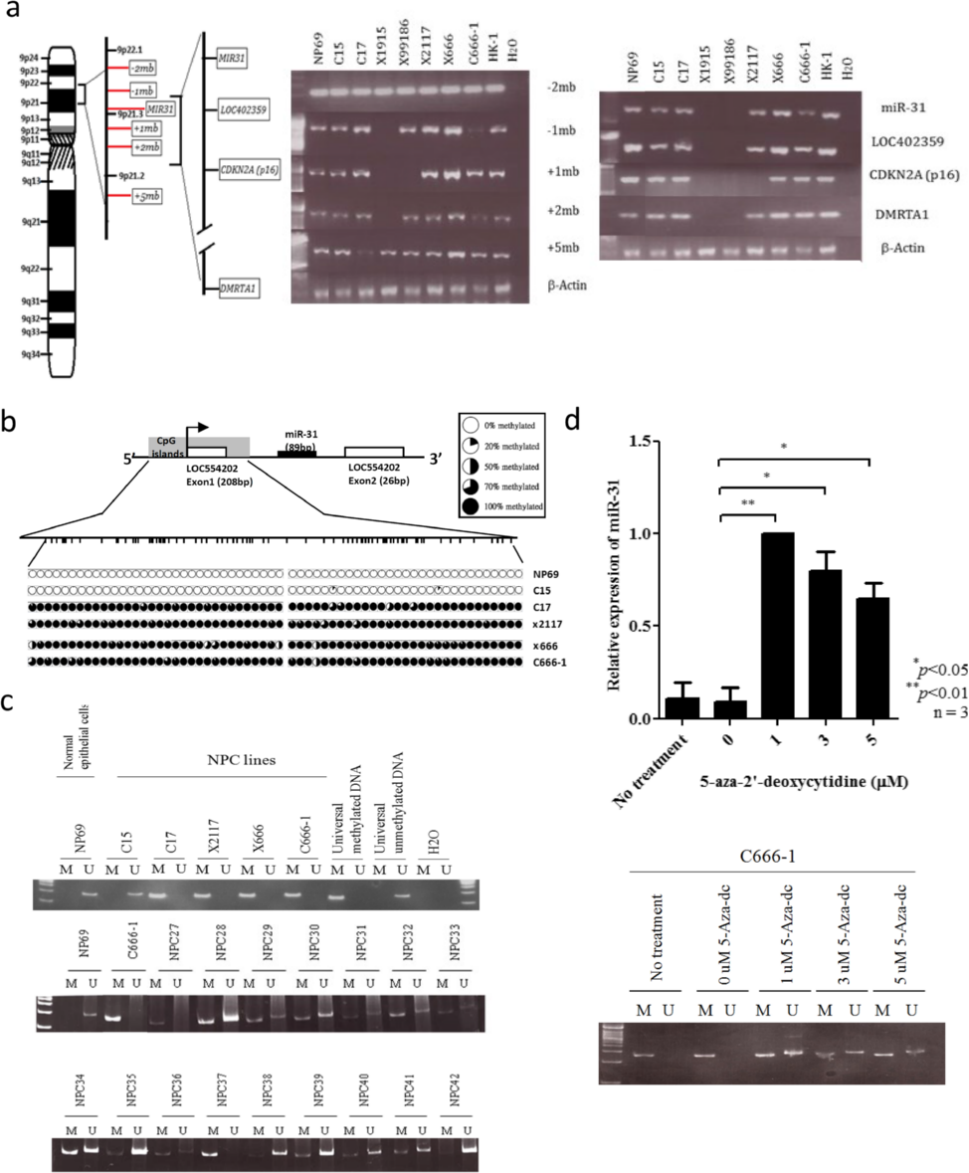
**Inactivation of *miR-31 *in NPC. (a)** Homozygous deletions of *miR-31*, adjacent markers (−2mb, −1mb, +1mb, +2mb, +5mb) and loci (*LOC402359*, *CDKN2A/p16*, *DMRTA1*) in EBV-positive NPC tumor lines were detected by PCR. The location of *miR-31* and adjacent markers on chromosome 9p21.3 was shown in the right panel. Homozygous deletions of *miR-31* were detected in X1915 and X99186. **(b)** Methylation status of 5’ CpG islands of *miR-31* in NPC tumor lines was examined by bisulfite sequencing. The locations of *miR-31* and its host gene *LOC554202* were indicated. Dense methylation of 5’CpG islands were detected in the C666-1 cell lines and 3 xenografts (C17, X2117 and X666). No methylation were observed in the normal control, NP69. **(c)** Detection of promoter hypermethylation of NPC cell lines and primary tumors by MSP. M: methylated allele; U: unmethylated alleles. **(d)** The restoration of *miR-31* transcription in 5’-aza-2’deoxycytidine (5-Aza-dC) treated C666-1 was detected by quantitative RT-PCR analysis. By MSP, unmethylated alleles of *miR-31* were found in the 5-Aza-dC-treated C666-1 cells.

Homozygous deletion of *p16/CDNK2B* locus on 9p21.3 was previously reported and identified by aCGH analysis in 3 PDXs (xeno-2117, xeno-1915 and xeno-99186) (Additional file 2: Figure S2) [11,16]. It is suspected that the down-regulation of *miR-31* in these tumors is due to complete loss of the *miR-31* allele. However, detailed mapping of the deletion regions by multiple PCR analysis demonstrated that *miR-31* locus was deleted in only 2 out of 6 xenografts (33.3%; xeno-1915 and xeno-99186) (Figure 2a). The expression of *miR-31* was regulated by the promoter of its host gene *LOC554202* (Figure 2a). Hypermethylation of the *LOC554202*-associated 5’CpG islands can lead to the transcription silencing of *miR-31*[17]. In the 4 NPC tumor lines with *miR-31* down-regulation, heavy methylation of the 5’CpG islands was detected by bisulfite sequencing and methylation-specific PCR (Figure 2b and 2c). Notably, promoter hypermethylation of *miR-31* was commonly found in the primary tumors (14/16; 87%) (Figure 2b). As shown in Figure 2d, re-expression of *miR-31* and unmethylated allele were detected in the C666-1 cells treated with a DNA methylation inhibitor, 5’-aza-2’deoxycytidine (5-Aza-dC). The findings indicated that homozygous deletion and methylation of 5’ CpG islands are the major mechanisms for *miR-31* inactivation in NPC.

### *miR-31* inhibits cell proliferation, viability and migration in NPC cells

**Figure 3:**
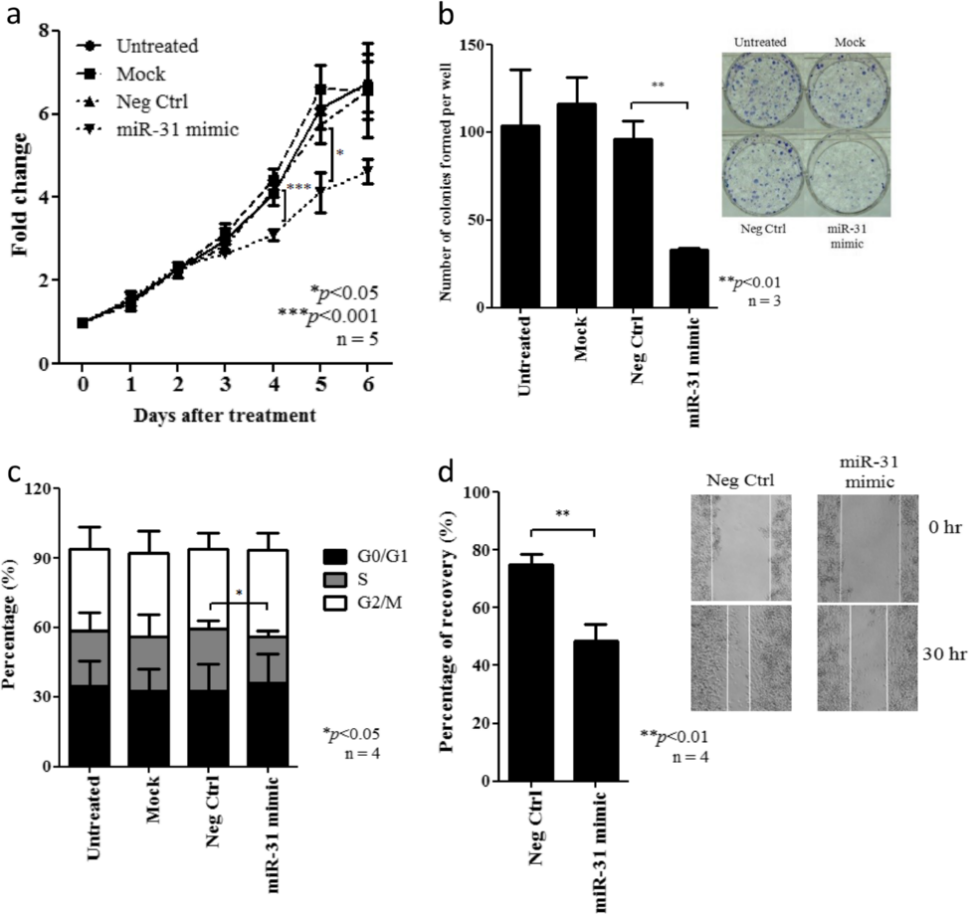
**Effect of *miR-31 *expression on cell growth and migration of NPC cells. (a)** By WST-1 assay, significant growth inhibition was detected in the C666-1 cell transfected with *miR-31* when compared with negative control. Data shown were taken from 5 independent experiments with mean ± SEM. **(b)** Expression of *miR-31* significantly inhibited the colony-forming ability of C666-1 cells (**p < 0.01). Representative photographs of colonies formed in each treatment were shown. Colonies formed were stained in blue by Giemsa stain. **(c)** Flow cytometry analysis revealed significant reduction of the percentage of cells undergoing S phase in *miR-31*-transfected C666-1 cells (*p < 0.05). **(d)** By wound healing assay, significant reduction of the migration ability of *miR-31*-expressing C666-1 (**P < 0.01). Representative photographs of wound healing progress of C666-1 cells transfected with negative control and miR-31 at 0 hour and 30 hours were shown.

To explore the tumor suppressor function of *miR-31* in NPC cells, the C666-1 cells, in which *miR-31* transcripts are downregulated, were transiently transfected with miR*-31* mimic or corresponding control. By WST-1 assay, we demonstrated that ectopic expression of *miR-31* significantly inhibited the cell proliferation and viability of C666-1 cells (Figure 3a). The *miR-31* expression also suppressed the clone formation ability of NPC cells. The number of colonies significantly reduced by 66% in *miR-31*-transfected C666-1 when compared to that of negative control in the colony formation assay (Figure 3b). By flow cytometry, a significant decrease in the percentage of C666-1 cells undergoing S-phase in cell cycle was detected after transient transfection of *miR-31* mimic (Figure 3c). In addition, a decrease of 35% in wound recovery area was measured in *miR-31*-transfected C666-1 when compared to that of negative control (Figure 3d). This implied that *miR-31* expression also inhibited the migratory capacity of NPC cells.

### *miR-31* suppresses tumorigenicity *in vitro* and *in vivo*

**Figure 4:**
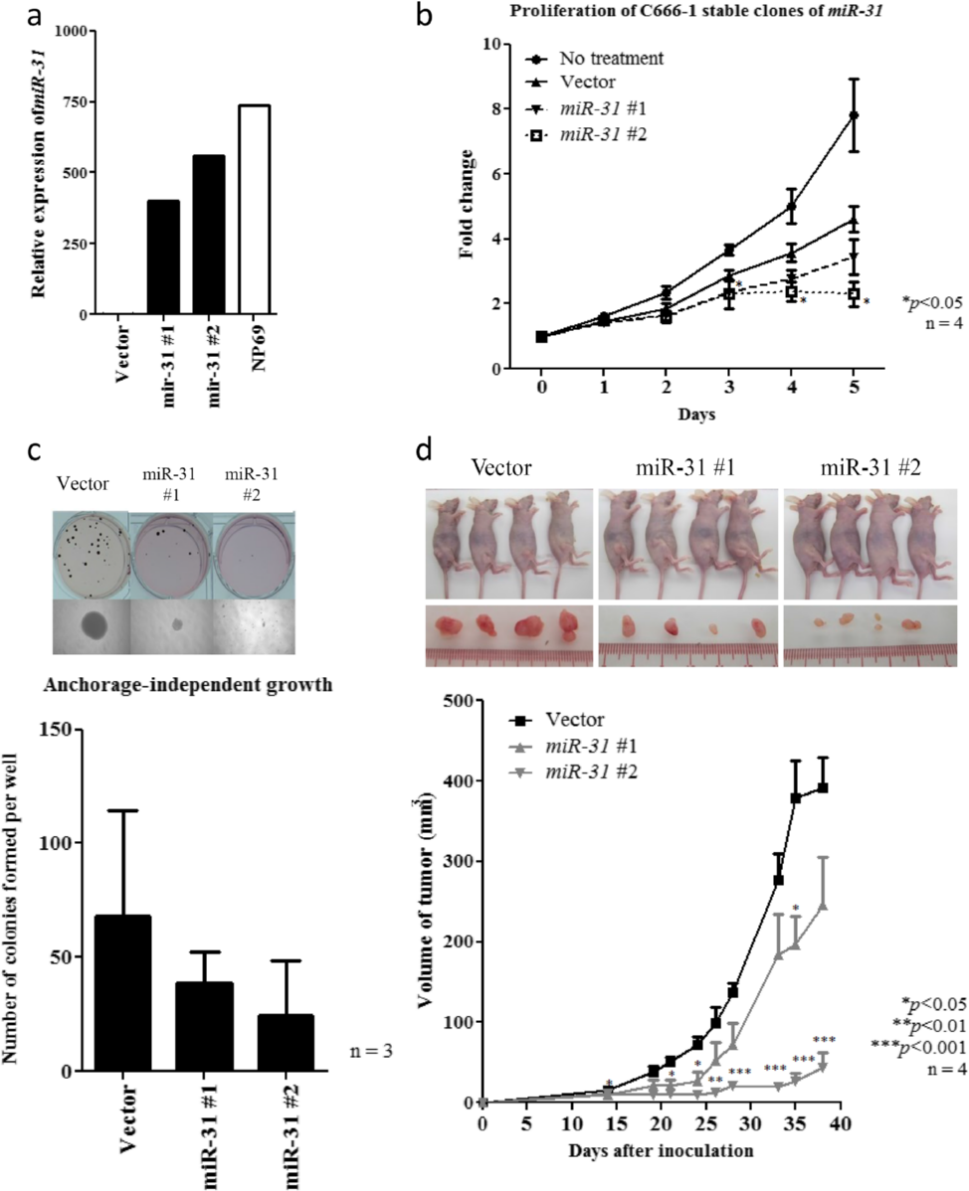
**Stable ectopic *miR-31 *expression suppresses the anchorage-independent growth and *in vivo *tumorigenicity of NPC cells. (a)** Expression of *miR-31* was demonstrated in the stably miR-31-transfected C666-1 cell clones (miR31#1 and miR31#2) by quantitative RT-PCR. The immortalized normal nasopharyngeal epithelial cells NP69 was included as control. **(b)** In the two stably *miR-31*-transfected NPC cell clones (miR31#1 and miR31#2), obvious growth inhibition was demonstrated by WST-1 assay. **(c)** Stable expression of *miR-31* inhibits the anchorage-independent growth of C666-1 cells. Obviously reduction in number and size of colonies in the stable *miR-31*-expressing cells were demonstrated by soft agar assay. **(d)***In vivo* tumorgenic assay in nude mice showed that tumors formed in the sites implanted with C666-1 cells expressing *miR-31* (miR-31#1 and miR-31#2) were consistently smaller than those implanted with vector controls. Photographs showing the nude mice (upper row) inoculated with stable clones (vector, *miR-31* #1, #2) and tumors extracted (bottom row) on day 38 after inoculation were also shown. Four nude mice were used in the experiment and data was shown with mean ± SEM. Student-t test was used for statistical significance, with a p-value of less than 0.05 was considered significant (*p < 0.05, **p < 0.01, ***p < 0.001).

To further explore whether ectopic expression of *miR-31* affects anchorage-independent growth *in vitro* and tumor growth *in vivo*, we have established 2 stably transfected C666-1 cell lines (miR-31#1 and miR-31#2) expressing different amount of *miR31* (Figure 4a). As shown in Figure 4b, significant suppression in cell proliferation was confirmed in both two stably-*miR-31* expressing cells. The stably transfected C666-1 cells with *miR-31* showed obvious repression of anchorage-independent growth. The cells expressing *miR-31* displayed much fewer and smaller colonies in the soft agar compared with controls (Figure 4c). To investigate the effect of *miR-31* on *in vivo* tumor growth, the stably *miR-31*-expressing C666-1 cells and controls were subcutaneously injected into nude mice. As shown in Figure 4d, tumor growth by *miR-31*-expressing cells was significantly inhibited when compared to those transfected with control vector. Notably, the stronger inhibitory effects on *in vitro* and *in vivo* tumor growth were found in the miR-31#2 clone which expressed higher level of *miR-31*. Our study demonstrated a dose-dependent tumor suppressor effect of *miR-31* in NPC cells.

### MCM2 and FIH1 as target of *miR-31* in NPC

**Figure 5:**
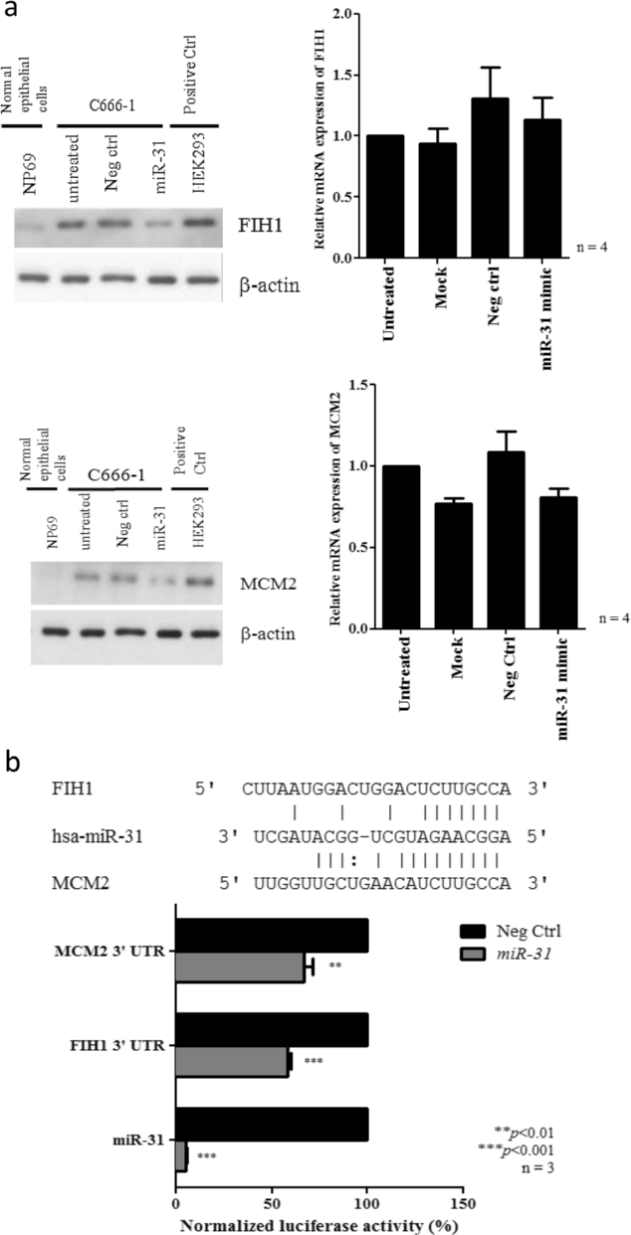
***miR-31 *suppresses FIH1 and MCM2 expression in NPC cells. (a)** Protein expression of FIH1 and MCM2 proteins were reduced in the C666-1 cells transfected with miR-31 when compared with controls (Right panel). By qRT-PCR, no significant changes of *FIH1* and *MCM2* transcripts were found in the miR-31-transfected C666-1 cells (Left panel). **(b)** Luciferase reporter assay showing the effects of *miR-31* on 3’ untranslated region (3’UTR) of *FIH1* and *MCM2* mRNA. Luciferase activity was normalized by the renilla luciferase control. The binding of *miR-31* on 3’UTR of *FIH1* and *MCM2* significantly decreased the luciferase activity. As a control, reporter vector carrying *miR-31* complementary sequence in the 3’ UTR were also constructed (*miR-31*) which upon binding showed a near complete abolishment of *miR-31* luciferase activity. Three independent experiments with mean ± SEM. Student-t test was used for statistical significance, with a *p*-value of less than 0.05 was considered significant (**p < 0.01, ***p < 0.001).

**Figure 6:**
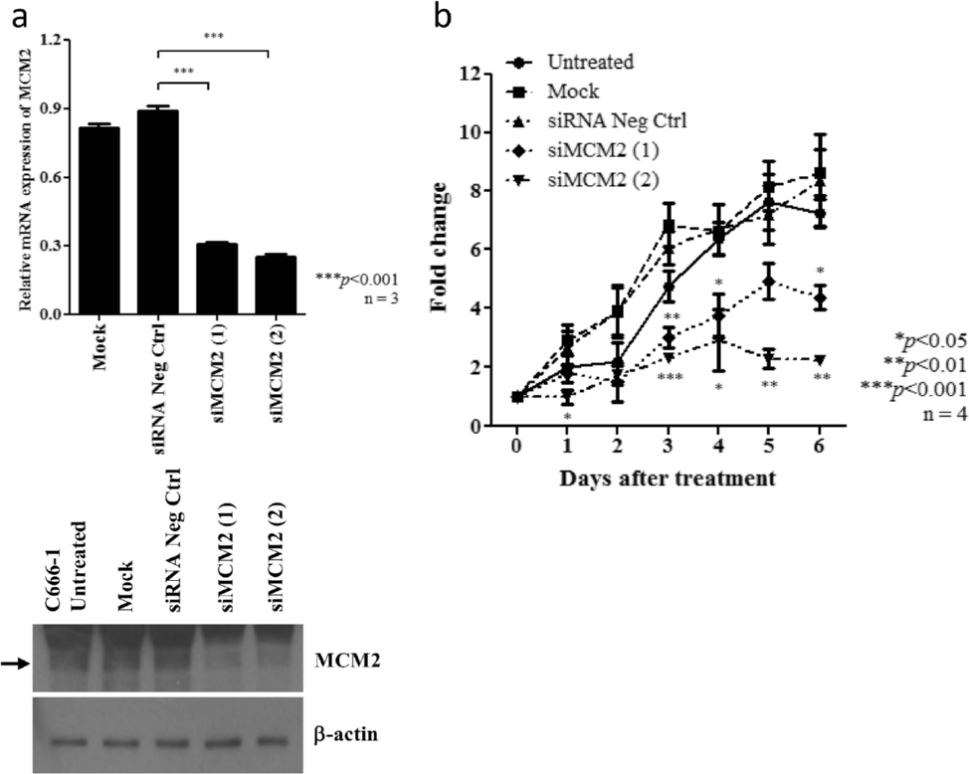
**Knock-down of MCM2 inhibits cell proliferation of NPC cells. (a)** Expression of MCM2 was knocked down by siRNAs targeting *MCM2* (siMCM2#1 and siMCM2#2). The suppression of MCM2 in C666-1 cells was confirmed by quantitative RT-PCR and Western blotting. **(b)** WST-1 assay demonstrated that cell proliferation was reduced in C666-1 cells treated with *MCM2* siRNAs (siMCM2#1 and siMCM2#2).

**Figure 7:**
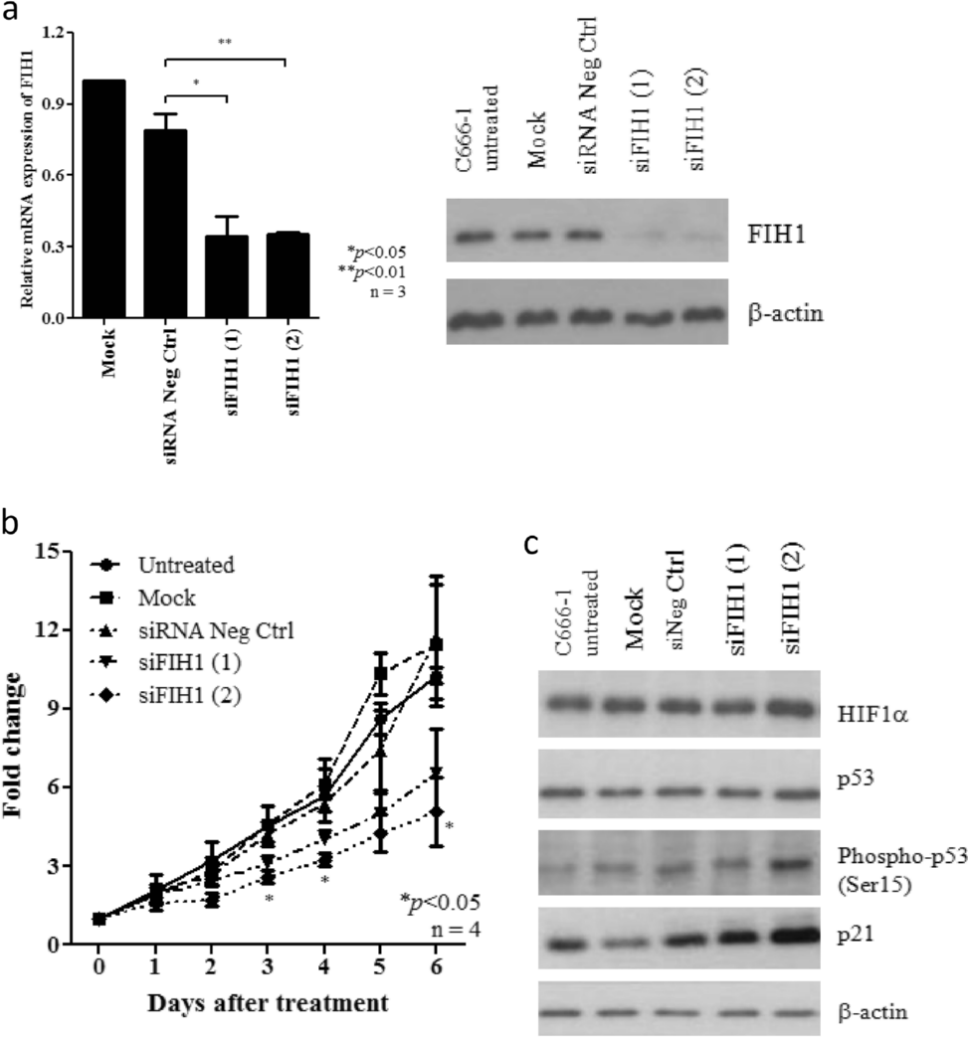
**Knock-down of FIH1 inhibits cell proliferation and enhances p53 phosphorylation in NPC cells. (a)** Expression of FIH1 was knocked down by siRNAs targeting *FIH1* (siFIH1#1 and siFIH1#2). The suppression of FIH1 in C666-1 cells was confirmed by quantitative RT-PCR and Western blotting. **(b)** WST-1 assay demonstrated that cell proliferation was reduced in C666-1 cells treated with *FIH1* siRNAs (siFIH1#1 and siFIH1#2). **(c)** By Western blotting, increase of phosphorylated p53 and p21 in C666-1 treated with *FIH1* siRNAs was demonstrated. Knock-down of FIH1 did not induce the expression of HIFα in NPC cells.

To investigate the mechanism by which *miR-31* suppressed the tumor cell growth in NPC, we validated a number of candidate targets of *miR-31* which are reported previously or predicated by the TargetScan and miRanda database. We found that *miR-31* expressed did not inhibit the expression of NIK, E2F2, RDX, RhoA, MCM7 in C666-1 cells (Additional file 3: Figure S3). Only FIH1 and MCM2 expression was obviously repressed by the *miR-31* in NPC cells (Figure 5a). Overexpression of these two proteins was commonly found in the NPC tumor lines (Additional file 4: Figure S4). By luciferase reporter assay, FIH1 and MCM2 were further confirmed to be direct targets of *miR-31* in C666-1. The binding of *miR-31* to the 3’ UTR of these genes markedly inhibited luciferase activity (Figure 5b). As shown in Figure 5, *miR-31* highly suppressed the expression of MCM2 and FIH1 in NPC cells. The finding confirmed *FIH1* and *MCM2* are direct targets of *miR-31* in NPC. MCM2 is a well-known component of the minichromosome maintenance (MCM) proteins 2–7 complex which plays crucial roles in DNA replication licensing. The important role of MCM2 in tumorigenesis has also been demonstrated in our previous report [18]. In this study, we also knocked down the expression of MCM2 in NPC C666-1 cells by siRNAs (Figure 6a). Significant growth inhibition of the C666-1 cells with MCM2 depletion was observed (Figure 6b). It indicated that *miR-31* may modulate NPC cell growth via repressing MCM2 expression. To further explore whether FIH1 is the target associated with the tumor suppressor function of *miR-31*, we knocked down the expression of *FIH1* by siRNAs in C666-1 cells and assessed its effects on growth inhibition (Figure 7a). As shown in Figure 7b, by WST-1 assay, the proliferation of C666-1 cells was significantly inhibited by the treatment of siRNAs targeting *FIH1*. Furthermore, we also found that *FIH1* knockdown enhanced Ser15 phosphorylation of p53 and up-regulated p21 expression (Figure 7c). The finding confirmed FIH1 function in the suppression of p53 activity as reported previously [19]. Since a majority of NPC contains the wild-type *p53*, down-regulation of *miR-31* is believed to be an important mechanism for impairing *p53* tumor suppressor function in this EBV-associated cancer.

## Discussion

NPC is a distinctive type of head and neck cancer which is consistently associated with EBV infection and prominent lymphoplasmacytic infiltration. Based on the studies on premalignant lesions and invasive cancers, we have proposed a model of NPC tumorigenesis, in which EBV infection and inactivation of multiple tumor suppressors on chromosomes 3p and 9p play crucial roles in the initiation process. The inactivation of *RASSF1A* on 3p21.3 was shown to be an early event in NPC development [20]. On chromosome 9p, we have previously demonstrated the consistent inactivation of *p16* on 9p21.3 in the primary tumors and precancerous lesions. In addition to a loss of growth inhibitory effects, *p16* inactivation predisposed nasopharyngeal epithelial cells to persistent EBV latent infection [15]. *miR-31* is a cancer-associated microRNA at 0.5 Mb telomeric to the *p16* locus and commonly deleted in various human cancers including melanoma, mesothelioma and urothelial carcinoma [7,21,22]. In this study, we clearly demonstrated that *miR-31* is consistently inactivated in NPC by either homozygous deletion or promoter methylation. As shown in the xenografts, both *miR-31* and *p16* loci are located in the common homozygous deletion region of NPC (Figure 2a). Notably, loss of *miR-31* expression was also detected in the pre-invasive lesions although the dysplastic lesions are rare and only limited cases were studied. The findings suggest the crucial role of *miR-31* in early development of NPC.

*miR-31* acts as tumor suppressor in several human malignancies, such as ATL (adult T cell leukemia), gastric cancer, mesothelioma, and melanoma [7,12,13,23]. Ectopic expression of *miR-31* inhibited *in vitro* cell proliferation and *in vivo* tumor growth in prostate cancers [8,24]. In our study, *miR-31* was found capable of inhibiting NPC cell proliferation, anchorage-independent growth, cell migration, and *in vivo* tumor development. The tumor suppressor properties of *miR-31* in EBV-associated NPC were confirmed. The phenotypes resulting from the tumor suppressor miRNA is dependent on its target genes. Although a number of experimental validated *miR-31* target genes have been reported, it is likely that the targets vary from one tumor type to another. Here, we confirmed *MCM2* and *FIH1* as *miR-31* target genes in NPC cells. The growth inhibitory effect of *miR-31* in NPC via modulating of MCM2 and FIH1 expression was demonstrated. MCM2 is one of the six components of MCM protein complex which is important in the initiation of DNA replication. Regulation of MCM2 protein expression by *miR-31* was recently reported in prostate cancer [8]. Elevated expression of MCM proteins were detected in both dysplasia and malignancy of various tissues [18]. It is believed that deregulation of MCM proteins contribute to the early stage in carcinogenesis. In our earlier study, we demonstrated that knockdown of MCM2 significantly inhibited the cell growth, migration, and invasion in medulloblastoma [18]. Furthermore, the involvement of MCM2 in regulating filopodia and stress fiber formation through cdc42 and Rho activation respectively was shown. Through knocking down of MCM2 by siRNA, studies demonstrated that the MCM2 expression impaired the growth of the prostatic (LNCaP) and colon (HCT116) cancer cell lines [18,25,26]. Similar growth inhibition was also observed in the C666-1 cells with MCM2 depletion. Occurrence of inhibitory phenotypes in the *miR-31*-expressing NPC cells is suspected to be due to MCM2 repression. Aside from MCM2, the suppressive effect of *miR-31* on NPC tumor growth was also via repressing FIH1. Liu *et al.* first reported *miR-31* target *FIH1* and thereby activates the HIF pathway in HNSCC [27]. High FIH1 expression contributes to the development of colon carcinomas and melanoma through the suppression of the p53-p21 axis [19]. Interestingly, FIH1 overexpression is also sufficient to inhibit differentiation of primary human corneal epithelial keratinocytes (HCEKs) [28]. Knocking down of FIH1 suppressed the cell proliferation in the clear cell renal cell carcinoma (CCRCC) (RCC10 and RCC4) and colon adenocarcinoma (LS174) cell lines [19,29]. Silencing of *FIH1* results in the elevation of p53 activity and p21 expression under normoxia [19]. Here, we also showed an increase in the expression of phospho-p53 (Ser15) and p21 in the NPC C666-1 cells with *FIH1* silencing. Since *p53* mutation is rare in EBV-associated NPC, impaired p53 function may be associated with high FIH1 expression in this *miR-31* deficient cells. Although *miR-31* modulated the expression of FIH1 in NPC cells, it did not alter HIF1α expression as shown in head and neck squamous cell carcinoma (HNSCC) [27]. HIF1α expression in the C666-1 cells was also not affected by knockdown of *FIH1* (Figure 7e). The oncogenic function of *FIH1* in NPC cells is likely to be HIF-independent. EBV-encoded LMP1 is capable to upregulate HIF1α through inducing Siah1 E3 ubiquitin ligase which promotes the degradation of prolyl hydroxylases 1 and 3 in nasopharyngeal epithelial cells [30]. In our study, high *miR-31* expression was detected in the xenograft C15 which shows homogeneous LMP1 expression (Additional file 5: Figure S5). The observation raises the possibility of interplay between these two proteins in NPC. Such potential crosstalk of viral protein and cellular microRNA needs to be further investigate in future study.

## Conclusions

Our study revealed that miR-31 is frequently inactivated in NPC and its inactivation is believed to be an early event in tumorigenesis. *miR-31* may target *MCM2* and *FIH1* and thereby inhibit growth of NPC cells. The significant inhibitory effects of miR-31 on *in vitro* and *in vivo* tumorigenicity implied *miR-31* as a potential therapeutic target for EBV-associated NPC. Our findings provide important understanding for the further elucidation on the therapeutic use of miRNA in NPC.

## Materials and methods

### Cell lines, xenografts and primary tumors

Six NPC patient-derived xenografts (PDXs) (C15, C17, xeno-2117 (X2117), xeno-666 (X666), xeno-1915 (X1915), and xeno-99186 (X99186)), an EBV-positive NPC cell line (C666-1), and an immortalized normal nasopharynx epithelial cell line (NP69) established by us were used in this study [31-37]. The study also included a total of 37 NPC endoscopic biopsies and 3 normal nasopharyngeal epithelium specimens obtained from NPC patients in Prince of Wales Hospital, The Chinese University of Hong Kong with informed consent. All NPC specimens were taken before treatment and confirmed to be non-keratinizing carcinoma and EBV positive. To enrich the collection of tumor cells or normal nasopharyngeal epithelial cells, microdissection was conducted manually on these samples. DNA and RNA extraction were performed as previously reported [5]. The patient characteristics are listed in Additional file 6: Table S1.

To assess the involvement of promoter methylation in *miR-31* silencing, C666-1 cells at 30% confluence were treated with the demethylation agent 5-aza-2’deoxycytidine (5-Aza-dC; Sigma-Aldrich). Half of the medium was replaced with fresh complete medium containing 5-Aza-dC every day for 3 days. Cells were harvested on day 4 for DNA or RNA extraction.

### PCR and Quantitative RT-PCR

To delineate the 9p21.3 homozygous deletion region in NPC xenografts, conventional PCR analysis of the loci in this region was performed using multiple primer pairs. The sequences of these primers were listed in Additional file 7: Table S2.

Total RNA from homogenized xenografts and cell lines was extracted using TRIZOL® reagent (Life Technologies). Conventional qRT-PCR using SuperScript™ III Reverse Transcriptase (Life Technologies) was performed for the detection of mRNA expression of target genes as described [38]. For determining microRNA expression, TaqMan MicroRNA Assay (Life Technologies) was performed according to the manufacturer’s instructions. The assays employed pre-designed, target-specific stem-loop reverse transcription miRNA primers (Life Technologies) for the mature miRNAs.

### *In situ* hybridization (ISH) analysis

By using the miRCURY LNA microRNA ISH Optimization Kit (Exiqon), *in situ* hybridization was performed to access the miR-31 expression of on the NPC tumor and dysplastic lesions. Four micron paraffin-embedded tissue sections were incubated with proteinase-K buffer (Exiqon) at 37°C for 20 minutes after deparaffinization by xylene and rehydration by ethanol. The slides were subjected to hybridization at 55°C. After washing, the sections were counter-stained with methyl green.

### Bisulfite sequencing and Methylation specific-PCR (MSP) analysis

For examining the methylation status, the DNA samples were subjected to bisulfite modification using EZ DNA Methylation-Gold Kit (Zymo Research). The modified DNA was subjected to bisulfite sequencing and MSP analysis as described [39,40]. The primers used were listed in Additional file 7: Table S2.

### miR-31 mimic and siRNA transfection

C666-1 cells were transiently transfected with *miR-31* mimic or negative control (Life Technologies). The transfection was performed according to manufacturer’s instructions of Lipofectamine 2000 (Life Technologies). Stably transfected C666-1 cells were generated by G418 selection of the clone transfected with *miR-31* expressing vector and miR-negative control (Origene) for 40 days. To knock down the expression of *FIH1* and *MCM2*, two independent specific siRNA duplexes for each gene were transfected into C666-1 cells, using LipofectAMINE 2000 (Invitrogen, Carlsbad, CA, USA) as described (Additional file 8: Table S3) [41]. Non-specific control siRNA and reagent control were included in the experiments.

### Cell proliferation, colony formation and cell migration assays

Cell proliferation and anchorage-dependent growth of miR-31-transfected C666-1 cells was determined by performing WST-1 and colony-formation assays as previously described [34]. The cells were also fixed and stained with propidium iodide, and then subjected to flow cytometry analysis using BD FACS Calibur (Becton Dickinson) and FlowJo software (Treestar). The migration capability was determined using wound closure assay as described [42].

### Anchorage-independent growth and *in vivo* tumorigenicity assays

Stably miR-31-transfected C666-1 and control cells were subjected to the soft agar assay for anchorage-independent growth in 4 mL medium supplemented with 0.35% agarose and layered on a 5 mL base of 0.7% agarose [41]. Experiments were carried out in triplicate. After 40 days, cells were stained with 0.8 mM p-iodonitrotetrazolium violet (Sigma-Aldrich). The *in vivo* tumorigenicity assay was performed as described previously [41]. A total of 2 × 10^6^ C666-1 cells stably expressing miR-31 or controls were subcutaneously inoculated into the flank of female BALB/c nude mice (nu/nu) (4 mice/construct). Tumor growth was monitored and the tumors were excised at the end of the experiment. All experimental procedures were approved by the Animal Ethics Committee of the Chinese University of Hong Kong.

### Luciferase reporter assay

C666-1 cells at 60% confluence in 96-well plate were co-transfected with *miR-31* mimic and reporter plasmid. After 48 hours of transfection, cells were lysed with 1X passive lysis buffer (Promega) at room temperature for 20 minutes. The lysates were then transferred to a 96-well ELISA plate and enzyme activities were assayed using the Dual Luciferase Reporter Kit (Promega).

### Western blotting

By western blotting, the expression of various proteins in the miR-31 transfected and siRNA-treated NPC cells was detected. The antibodies against p21 Waf1/Cip1 (Abcam), Phospho-p53 (Ser15) (Abcam), HIF1α (Abcam), FIH1 (Santa Cruz), MCM2 (Santa Cruz) and ACTIN (Santa Cruz) were used.

## Abbreviations

NPC: Nasopharyngeal carcinoma; EBV: Epstein-Barr virus; X666: Xeno-666; X2117: Xeno-2117; X1915: Xeno-1915; X99186: Xeno-99186; miRNA: microRNA.

## Competing interests

The authors declare that they have no competing interests.

## Authors’ contributions

KWL, CCMC, and GTYC designed the study; CCMC, GTYC, SWML, KWC, SPS and KML carried out experiments; KFT, RKCN, TTCY, PB, XYG, SWT provided the NPC tumor models, primary tumor specimens and clinical data; KWL, KFT, CCMC , GTYC, SWML were involved in data analysis and writing the paper. All authors had final approval of the submitted manuscript.

## Supplementary Material

Additional file 1: Figure S1 Heat map of expression profiles of differentially expressed miRNAs in immortalized normal epithelial cell line NP69 and NPC tumor lines. The normalized data was log2-transformed and each miRNA was scaled among all the samples. Hierarchical clustering with average linkage algorithm and using one-minus correlation for determination of similarity was performed to cluster the samples and miRNAs. High expression is depicted as red while green box represents low expression. Click here for file

Additional file 2: Figure S2 High resolution array-CGH analysis of chromosome 9p21 region in three NPC xenografts, (a) xeno-99186, (b) xeno-1915, and (c) xeno-2117. The locations of miR-31, CDKN2A/p16 and DMRTA1 are indicated. Click here for file

Additional file 3: Figure S3 Ectopic expression of miR-31 did not suppress the expression of NIK, E2F2, RDX, RhoA and MCM7 in NPC cells. By western blotting, no significant reduction of several reported *miR-31* targets including NIK, E2F2, RDX, RhoA and MCM7 were detected in *miR-*31-transfected C666-1 cells. Click here for file

Additional file 4: Figure S4 Overexpression of MCM2 and FIH1 in NPC tumor lines. By western blotting, high MCM2 and FIH1 expression were detected in C666-1 and the xenografts. Weak expression of both MCM and FIH1 were found in the immortalized nasopharyngeal epithelial cells NP69. Click here for file

Additional file 5: Figure S5 LMP1 expression in NPC cell line and xenografts. The expression of LMP1 in NPC cell line and xenografts was determined by quantitative RT-PCR. By immunohistochemistry staining, LMP1 expression in C15, C17 and xeno-2117 was shown. The assays were performed as we previously described [43]. Click here for file

Additional file 6: Table S1 The characteristics of NPC patients involved in this study. Click here for file

Additional file 7: Table S2 List of primer sequences used in PCR, qRT-PCR, MSP and bisufite sequencing analysis. Click here for file

Additional file 8: Table S3 List of siRNA sequences used in this study. Click here for file
