# Supplementary figures and images for: miR-31 is consistently inactivated in EBV-associated nasopharyngeal carcinoma and contributes to its tumorigenesis
Source: Mol Cancer. 2014 Aug 7;13:184. doi: 10.1186/1476-4598-13-184 (PMC4127521; doi:10.1186/1476-4598-13-184)

Figure S1

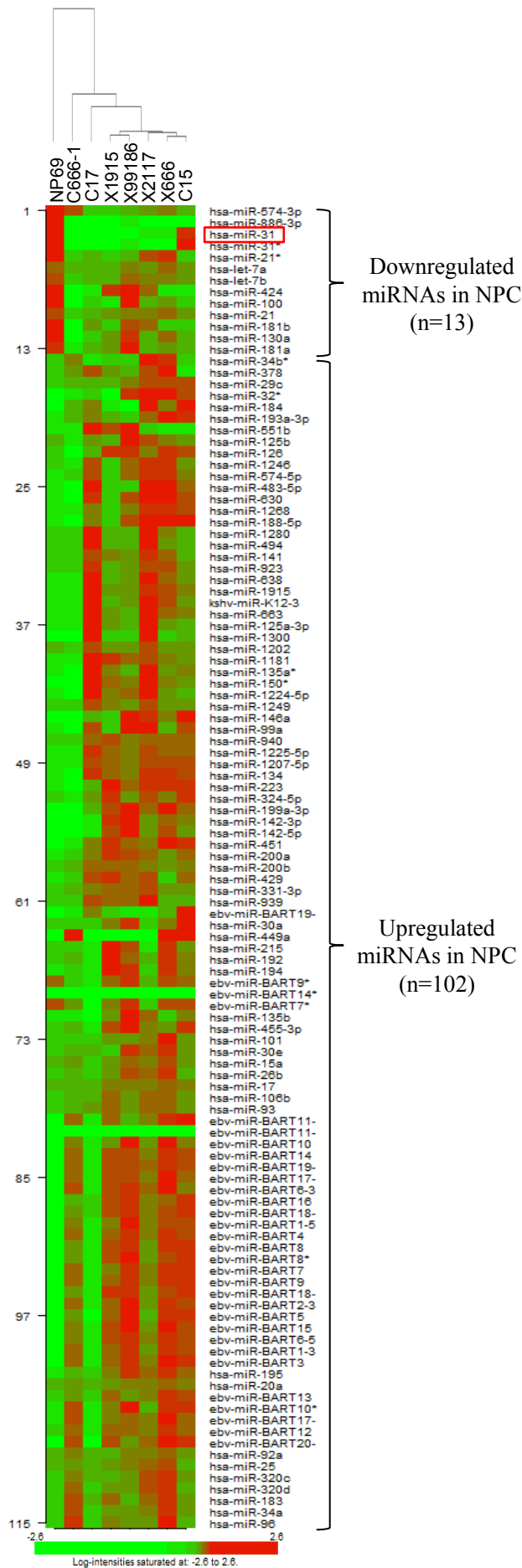

Supplement: Additional file 1: Figure S1 — Heat map of expression profiles of differentially expressed miRNAs in immortalized normal epithelial cell line NP69 and NPC tumor lines. The normalized data was log2-transformed and each miRNA was scaled among all the samples. Hierarchical clustering with average linkage algorithm and using one-minus correlation for determination of similarity was performed to cluster the samples and miRNAs. High expression is depicted as red while green box represents low expression. [file 1476-4598-13-184-S1.pdf]

Figure S2

A

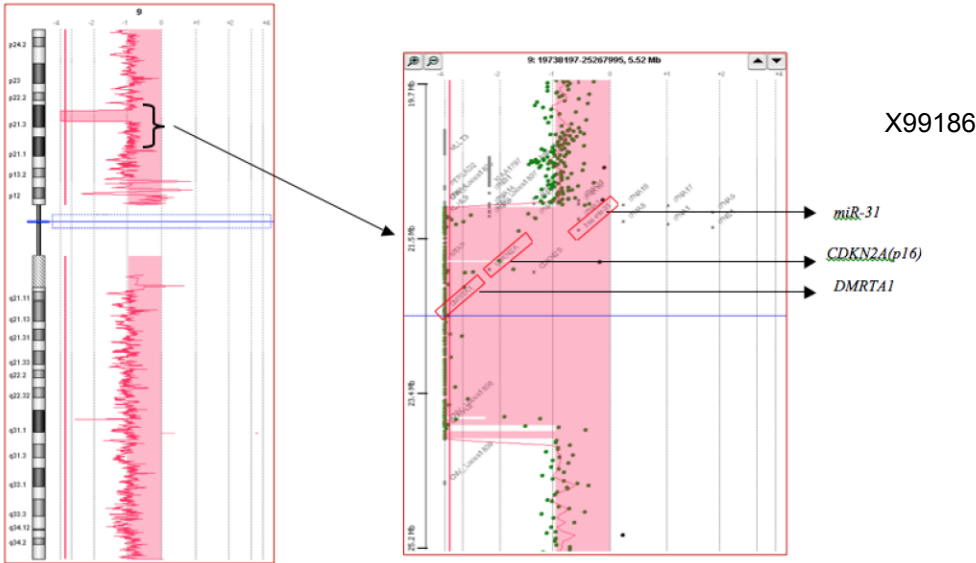

B

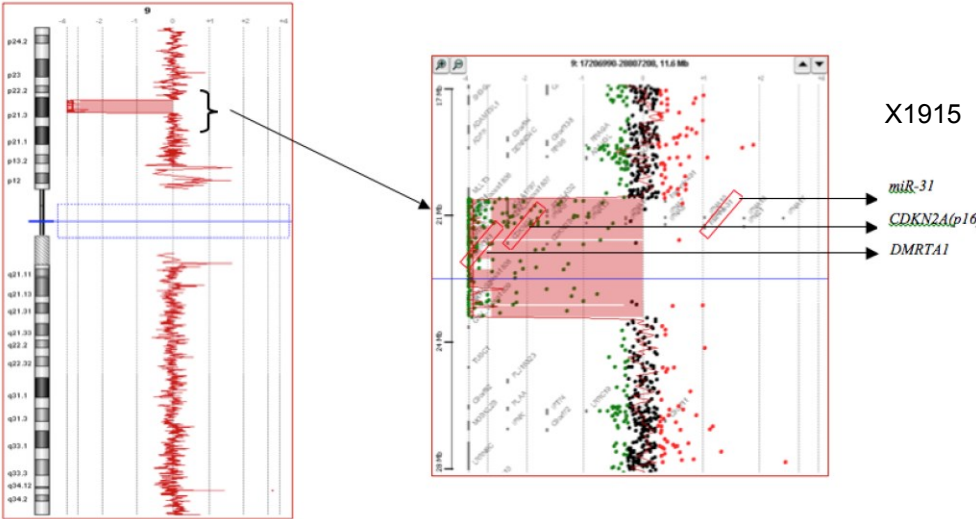

C

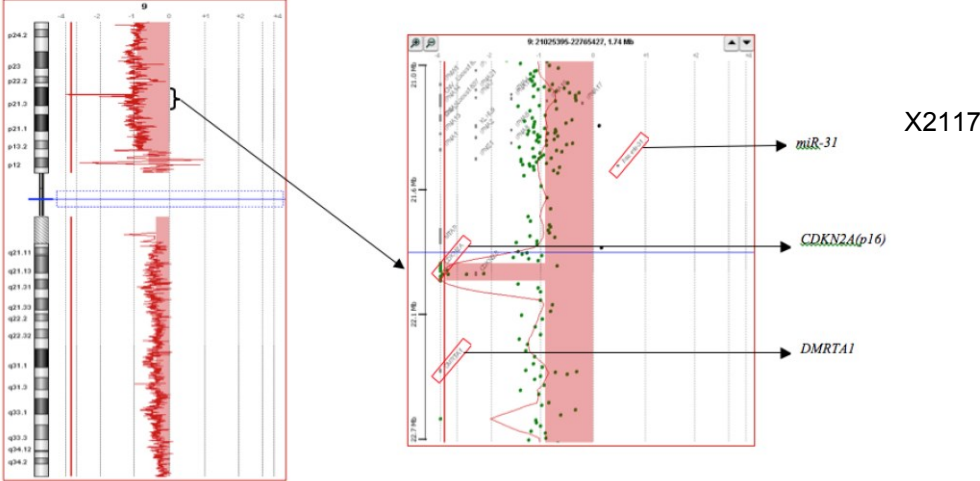

Supplement: Additional file 2: Figure S2 — High resolution array-CGH analysis of chromosome 9p21 region in three NPC xenografts, (a) xeno-99186, (b) xeno-1915, and (c) xeno-2117. The locations of miR-31, CDKN2A/p16 and DMRTA1 are indicated. [file 1476-4598-13-184-S2.pdf]

Figure S3

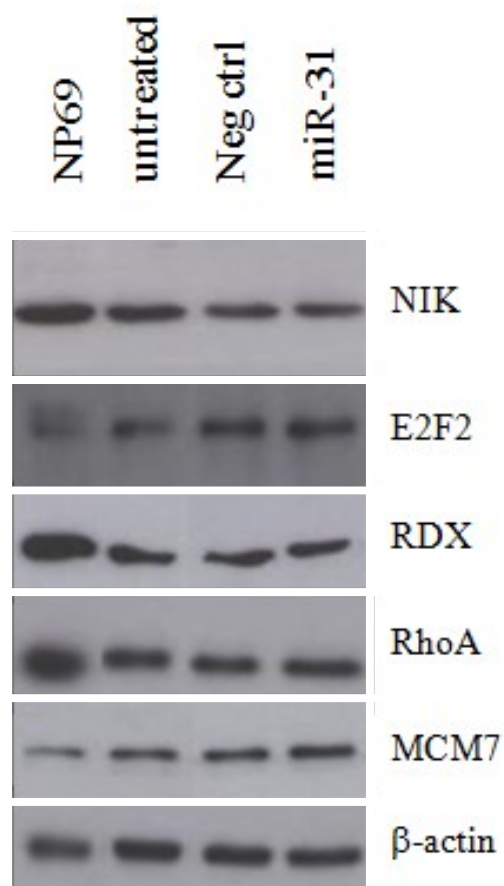

Supplement: Additional file 3: Figure S3 — Ectopic expression of miR-31 did not suppress the expression of NIK, E2F2, RDX, RhoA and MCM7 in NPC cells. By western blotting, no significant reduction of several reported miR-31 targets including NIK, E2F2, RDX, RhoA and MCM7 were detected in miR-31-transfected C666-1 cells. [file 1476-4598-13-184-S3.pdf]

Figure S4

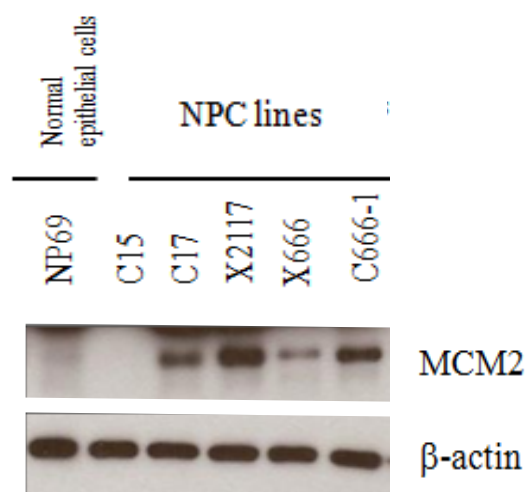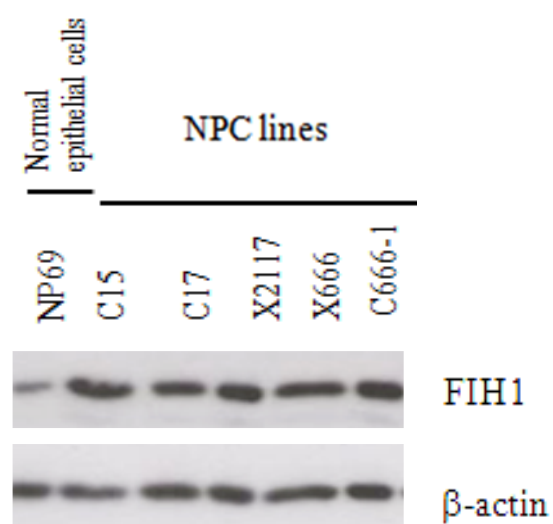

Supplement: Additional file 4: Figure S4 — Overexpression of MCM2 and FIH1 in NPC tumor lines. By western blotting, high MCM2 and FIH1 expression were detected in C666-1 and the xenografts. Weak expression of both MCM and FIH1 were found in the immortalized nasopharyngeal epithelial cells NP69. [file 1476-4598-13-184-S4.pdf]
