# Supplementary material for: miR-31 is consistently inactivated in EBV-associated nasopharyngeal carcinoma and contributes to its tumorigenesis
Source: Mol Cancer. 2014 Aug 7;13:184. doi: 10.1186/1476-4598-13-184 (PMC4127521; doi:10.1186/1476-4598-13-184)
Supplement: Additional file 5: Figure S5 — LMP1 expression in NPC cell line and xenografts. The expression of LMP1 in NPC cell line and xenografts was determined by quantitative RT-PCR. By immunohistochemistry staining, LMP1 expression in C15, C17 and xeno-2117 was shown. The assays were performed as we previously described [43]. [file 1476-4598-13-184-S5.pdf]

Figure S5

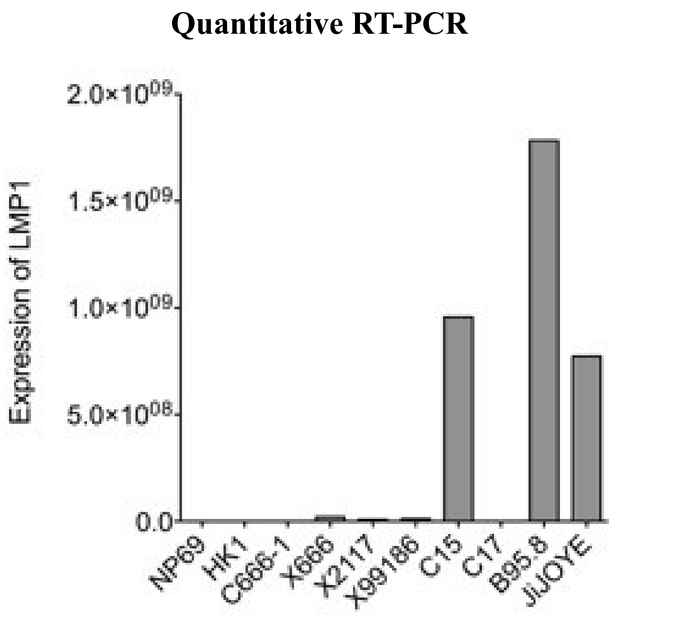

Expression of LMP1 in EBV-positive tumor lines

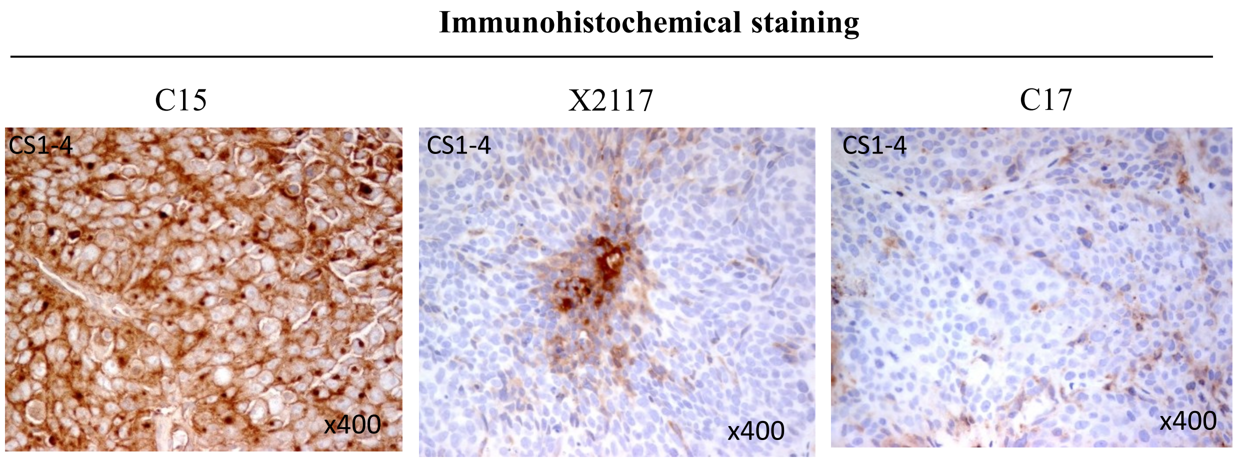

LMP1 protein expression in EBV-positive NPC tumor lines
