# Supplementary material for: miR-31 is consistently inactivated in EBV-associated nasopharyngeal carcinoma and contributes to its tumorigenesis
Source: Mol Cancer. 2014 Aug 7;13:184. doi: 10.1186/1476-4598-13-184 (PMC4127521; doi:10.1186/1476-4598-13-184)
Supplement: Additional file 6: Table S1 — The characteristics of NPC patients involved in this study. [file 1476-4598-13-184-S6.pdf]

Table S1. The characteristics of NPC patients involved in this study.

Characteristics of patients	n (%)
**No. of evaluable patients**	37
**Gender**	
Female	8 (21.6%)
Male	29 (78.4%)
**Age**	
Mean	50
Range	31-80
**Tumor stage**	
I	2 (5.4%)
II	17 (45.9%)
III	10 (27.0%)
IV	6 (16.2%)
