# Supplementary material for: miR-31 is consistently inactivated in EBV-associated nasopharyngeal carcinoma and contributes to its tumorigenesis
Source: Mol Cancer. 2014 Aug 7;13:184. doi: 10.1186/1476-4598-13-184 (PMC4127521; doi:10.1186/1476-4598-13-184)
Supplement: Additional file 7: Table S2 — List of primer sequences used in PCR, qRT-PCR, MSP and bisufite sequencing analysis. [file 1476-4598-13-184-S7.pdf]

Table S2. List of primer sequences used in PCR, qRT-PCR, MSP and bisulfite sequencing analysis.

**qRT-PCR primers**	**Sequence (5' → 3')**
HSA-MIR-31	Stem-loop:<br>GGAGAGGAGGCAAGAUGCUGGCAUA<br>GCUGUUGAACUGGGAACCUGCUAUGC<br>CAACAUAUUGCCAUCUUUCC
MCM2-F	CAAGACTTTTGCCCGCTACCT
MCM2-R	TCTGCCACTAACTGCTTCAGTATGA
FIH1-F	CACATAGGCTTGCGTCTTAAAGC
FIH1-R	TTGAGGGTGTTGTGGCACATT

**PCR primers for mapping**	**Sequence (5' → 3')**
9p – 1Mb-F	CCTGCTGTGCACTAGATCTC
9p – 1Mb-R	AGGGCTCCCACTGATTCTG
9p + 1Mb-F	GTTATGCTGCATTCCAGATGG
9p + 1Mb-R	GTAATGCTTCCAGGTCTATGC
9p – 2Mb-F	CTGCACGCGTTAACTCGTCAT
9p – 2Mb-R	GGAAACCATCATTCTCAGCAAACT
9p + 2Mb-F	GAATGCTGCGGAGAAACATG
9p + 2Mb-R	CACTGTGCAGATAAAGGGAAC
9 + 5Mb-F	AAACGTAGCAGTCAGGAGGC
9 + 5Mb-R	TCAACAGCGGGAATAACACA
LOC402359-F	TGGGGATCCTTACAAAGTGC
LOC402359-R	CTTCGTGTAGTCCTGCTGCC
DMRTA1-F	AGTGTCCTCAAGACCAAGAGATCCT
DMRTA1-R	TGAAAGGCTGTGTTTTCCAGTTC
MIR-31-F	CTTGAGGGTCCTATGGAGTCA
MIR-31-R	GCCAGTCCTTCGTGTATTGC
CDKN2A (p16)-F	GAATCCCGTAGCTTCCCTAC
CDKN2A (p16)-R	CGGGTCCCGATTTAGAAGG

**MSP primers**	**Sequence (5' → 3')**
miR-31-Left M primer	TTGTGTATAATTTGGGGCGTC
miR-31 Right M primer	CCAACTTACCTACGAATCCGA
miR-31 Left U primer	TTGTGTATAATTTGGGGTGTTGT
miR-31 Right U primer	CTCCCAACTTACCTACAAATCCA

**Bisulfite sequencing primers**	**Sequence (5’ → 3’)**
miR-31-Left primer (fragment 1)	GGTTTTTTAGGAGGAGTTTGGT
miR-31-Right primer (fragment 1)	AAAACTCTCCCAACTTACCTAC
miR-31-Left primer (fragment 2)	GTAGGTAAGTTGGGAGAGTTTT
miR-31-Right primer (fragment 2)	ACCTAAAATTTAAACAAAAAAAA
