# Supplementary material for: miR-31 is consistently inactivated in EBV-associated nasopharyngeal carcinoma and contributes to its tumorigenesis
Source: Mol Cancer. 2014 Aug 7;13:184. doi: 10.1186/1476-4598-13-184 (PMC4127521; doi:10.1186/1476-4598-13-184)
Supplement: Additional file 8: Table S3 — List of siRNA sequences used in this study. [file 1476-4598-13-184-S8.pdf]

Table S3. List of siRNA sequences

**siRNA**	**Sequence**
HIF1AN (FIH1)#1	CCCGACTACGAGAGGTTCCCTAATT
HIF1AN (FIH1)#2	GCTCATCAGAAAGTGGCCATAATGA
MCM2 #1	GGTCAACATGGAGGAGACCATCTAT
MCM2 #2	AGGACACTATTGAGGTCCCTGAGAA
